# Ferroptosis Susceptibility in Pediatric Immune Thrombocytopenia: Alteration in Mesenchymal Stromal Cells

**DOI:** 10.3390/ijms27198917

**Published:** 2026-10-08

**Authors:** Giuseppe Di Feo, Maria Maddalena Marrapodi, Oriana Di Domenico, Lucia Argenziano, Michele La Manna, Elisabetta Lembo, Giuseppe Palumbo, Francesca Rossi, Alessandra Di Paola

**Affiliations:** 1 Department of Woman, Child and General and Specialist Surgery, University of Campania “Luigi Vanvitelli”, 80138 Napoli, Italy; giuseppe.difeo.bio@gmail.com (G.D.F.); mariamaddalena.marrapodi@unicampania.it (M.M.M.); didomenico.oriana.odd@gmail.com (O.D.D.); lucia.argenziano@unicampania.it (L.A.); alessandra.dipaola@unicampania.it or a.dipaola@unilink.it (A.D.P.); 2 Department of Haematology, Bambino Gesù Hospital, 00165 Rome, Italy; michele.lamanna@opbg.net (M.L.M.); elisabetta.lembo@opbg.net (E.L.); giuseppe.palumbo@opbg.net (G.P.); 3 Department of Life Science, Health and Health Professions, Link Campus University, 00165 Rome, Italy

**Keywords:** ITP, ferroptosis, MSCs, iron metabolism, ROS

## Abstract

Immune thrombocytopenia (ITP) is the most common pediatric thrombocytopenia, characterized by immune-mediated platelet destruction and altered inflammatory responses. Among these alterations, mesenchymal stromal cells (MSCs) from ITP patients display reduced viability and impaired immunoregulatory functions. Our previous work revealed dysregulated iron metabolism in ITP-MSCs, with increased intracellular iron accumulation driven by hepcidin–FPN1 axis disruption and overexpression of iron importers. Given the link between iron overload, oxidative stress, and ferroptosis, a regulated iron-dependent form of cell death, we investigated whether ITP-MSCs exhibit oxidative and molecular alterations associated with ferroptosis. ITP-MSCs showed increased ROS levels and ACSL4 expression, together with reduced expression of proteins involved in ferroptosis-related antioxidant defense, including SLC3A2, SLC7A11, and GPX4. Increased extracellular release of HMGB1, a DAMP associated with cellular stress and different forms of regulated cell death, was also observed. Treatment with Ferrostatin-1 (Fer-1) and Deferasirox (DFX) reduced oxidative stress and partially restored the expression of ferroptosis-related markers. Similarly, high-dose dexamethasone (HD-Dexa) reduced ROS and ACSL4 levels and limited extracellular HMGB1 release. Collectively, these findings support increased susceptibility to ferroptotic stress in ITP-MSCs and reveal a ferroptosis-associated oxidative stress phenotype with concomitant alterations in ferroptosis-related molecular pathways. Moreover, the effects of HD-Dexa, DFX, and Fer-1 suggest that modulation of these pathways may represent a potential strategy for limiting oxidative cellular damage in ITP-MSCs.

## 1. Introduction

Immune thrombocytopenia (ITP) is the most common pediatric thrombocytopenia caused by autoantibody-mediated platelet destruction [1,2,3,4]. It is characterized by a platelet count of less than 100 × 10^9^/L [3]. Clinical manifestations can range from mild bleeding —including bruising and petechiae—to severe bleeding, which occurs in approximately 5% of patients [2,3,5,6,7].

ITP is characterized by dysregulated inflammatory and immune responses, including a predominance of pro-inflammatory classically activated M1 macrophages, an increased T helper (Th) 1/Th2 ratio and impairment of mesenchymal stromal cells (MSCs) function and activity [1,2,4,8,9,10,11,12].

MSCs are non-hematopoietic multipotent stromal cells involved in the regulation of immune and inflammatory cell functions, and exhibit anti-inflammatory and immunosuppressive properties [13]. These functions are strongly impaired in ITP, where MSCs show altered morphology, reduced viability, and undergo senescence and apoptosis [2,4,12,14,15].

In 2022, we identified dysregulated iron metabolism in ITP MSCs, potentially contributing to their functional impairment [2]. We observed an increased intracellular iron concentration in ITP MSCs, driven by an inflammation-mediated disruption of the hepcidin-ferroportin (FPN1) axis and by elevated expression of iron importers, including transferrin receptor 1 (TfR1) and divalent metal transporter 1 (DMT1) [2].

Iron and inflammation are strongly correlated, since iron accumulation can promote cell damage and exacerbate inflammatory processes [16], causing reactive oxygen species (ROS) production via the Fenton reaction, resulting in oxidative damage to cellular DNA, lipids, and proteins [17]. ROS-induced lipid damage deserves special attention due to its biological implications [17,18,19,20,21]. The main targets are represented by membrane phospholipids, especially polyunsaturated fatty acids (PUFAs), which confer fluidity and deformability to membranes [17,22]. PUFAs are highly susceptible to oxidation by ROS, resulting in lipid peroxidation. Acyl-CoA synthetase long-chain family member 4 (ACSL4) is an enzyme responsible for PUFA esterification into membrane phospholipids, by adding CoA to the long chain, leading to lipoxygenase (ALOX)-dependent lipid peroxidation [20,21,23,24]. The resulting lipid radicals interact with molecular oxygen present within the membrane, forming peroxidized-free radicals, which target membrane proteins and PUFAs, amplifying the lipid peroxidation cascade and compromising membrane integrity, leading to ferroptosis [17,18,19,21]. Ferroptosis, introduced for the first time by Dixon et al. in 2012 [25], is an iron-dependent, non-apoptotic form of regulated cell death, caused by iron accumulation and lipid peroxidation [17,18,21,26].

Under physiological conditions, an antioxidant system protects cells from oxidative stress and ferroptosis [18,21,24]. The System Xc^−^ is an amino acid antiporter localized at the plasma membrane, constituted by two different subunits, SLC7A11 and SLC3A2, which internalize cystine and release glutamate in a ratio of 1:1 [17,18]. Intracellular cystine is reduced to cysteine and contributes to glutathione (GSH) synthesis, which is essential for the glutathione peroxidases 4 (GPX4)-mediated reduction in phospholipid hydroperoxides [17,18,27,28]. During ferroptosis, cystine internalization is reduced, inhibiting GSH synthesis and determining GPX4 inactivation and the loss of cell antioxidant capacity, with increased levels of lipid ROS, cell oxidative damage and ferroptosis [17,18]. Moreover, ferroptotic cells release damage-associated molecular patterns (DAMPs), which promote immune and inflammatory responses. Among these, the high-mobility group box 1 (HMGB1) protein, normally located in the nucleus, can be released into the extracellular space during different forms of cell death, where it modulates immune and inflammatory processes [29].

Considering the alteration of iron metabolism and the consequent intracellular iron accumulation already reported in ITP MSCs [2], we hypothesized that ferroptosis-related pathways and oxidative stress may contribute to MSC dysfunction and increased susceptibility to ferroptotic stress in ITP.

In 2018, Rossi et al. demonstrated that high-dose dexamethasone (HD-Dexa), together with cannabinoid receptor 2 (CB2) stimulation, was able to restore MSCs activity and function, and ameliorate inflammatory state in ITP [4].

Therefore, we investigated oxidative stress and ferroptosis-related molecular alterations in ITP MSCs and evaluated their modulation by high-dose dexamethasone (HD-Dexa), deferasirox (DFX), and ferrostatin-1 (Fer-1). Specifically, we assessed ROS production and the expression of key proteins involved in ferroptosis-related pathways to explore whether these alterations may contribute to the dysfunctional phenotype previously described in ITP-MSCs.

## 2. Results

### 2.1. MSCs Characterization

Immunophenotype revealed no significant differences between MSCs derived from healthy donors (CTR-MSCs) and from ITP-MSCs. Both populations consistently exhibited high expression levels (100%) of surface markers CD105, CD73, CD90, and HLA-ABC, while lacking expression of hematopoietic (CD45, CD14, CD34, CD13) and endothelial (CD31) markers, and co-stimulatory molecules CD80 and HLA-DR (Supplementary Table S1). ITP-MSCs showed reduced proliferative capacity and appeared larger and more flattened compared to CTR-MSCs in vitro culture.

### 2.2. Effect of DFX and Fer-1 on MSCs Viability and Cytotoxicity

We performed an MTT assay to evaluate DFX and Fer-1 effects on MSCs viability and cytotoxicity. The results indicated DFX [1 µM], [5 µM] and [10 µM], and Fer-1 [1 µM] and [5 µM] did not impact the viability of MSCs compared to NT. Meanwhile, DFX [20 µM], [40 µM], [60 µM] and [80 µM], and Fer-1 [10 µM], [20 µM], [40 µM], [60 µM] and [80 µM] induced a strong reduction in cell viability, letting us consider these concentrations cytotoxic (Supplementary Tables S2 and S3).

### 2.3. Effect of HD-Dexa on ROS Production

**Figure 1:**
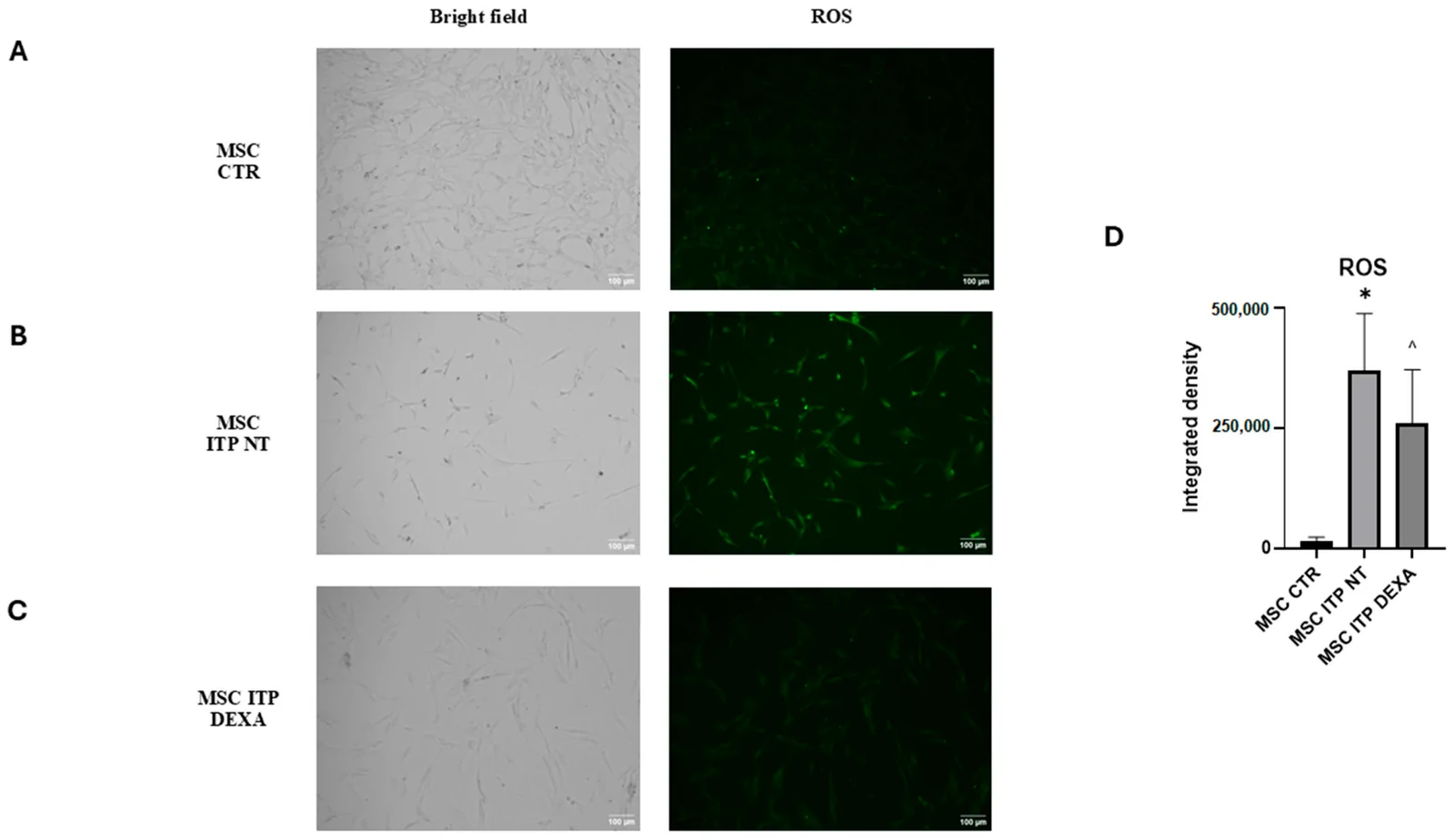
Evaluation of intracellular ROS in CTR MSCs and ITP MSCs at baseline (NT, 0 h) and in ITP MSCs after 24 h of High-dose dexamethasone treatment. ROS levels in mesenchymal stromal cells (MSCs) isolated from control (CTR) subjects (**A**) and immune thrombocytopenia (ITP) patients (**B**) before and after 24 h exposure to High-dose dexamethasone (HD-Dexa) [100 nM] (**C**). The most representative image is displayed. ROS were detected using Thermo Fisher EVOS M5000 Imaging System. ImageJ software (Fiji, version 1.52; NIH, Bethesda, MD, USA) was used to analyze the images, and integrated density was displayed (**D**). Representative images were acquired using a 10× objective. Scale bar: 100 µm. The graph represents the quantification as mean ± standard deviation (SD) of three independent biological samples per group (N = 3). A *t*-test has been used to evaluate statistical differences. *, *p* = 0.0067 compared to MSC CTR. ^ *p* = 0.0027 NT compared to MSC ITP NT.

To evaluate HD-Dexa effects in ITP-MSCs, we performed a ROS assay. We assessed ROS levels in both CTR-MSCs and ITP NT-MSCs, observing a higher percentage of ROS-positive cells in ITP NT-MSCs compared to CTR-MSCs (Figure 1A,B,D). Treatment with HD-Dexa [100 nM] decreased ROS production in ITP-MSCs compared to untreated cells (Figure 1B–D). These findings indicate that ITP-MSCs are characterized by excessive ROS production, which may contribute to their functional impairment. Furthermore, HD-Dexa [100 nM] appears to reduce ROS levels in ITP-MSCs, possibly by inhibiting the activation of ferroptosis.

### 2.4. Effect of DFX and Fer-1 on ROS Production

**Figure 2:**
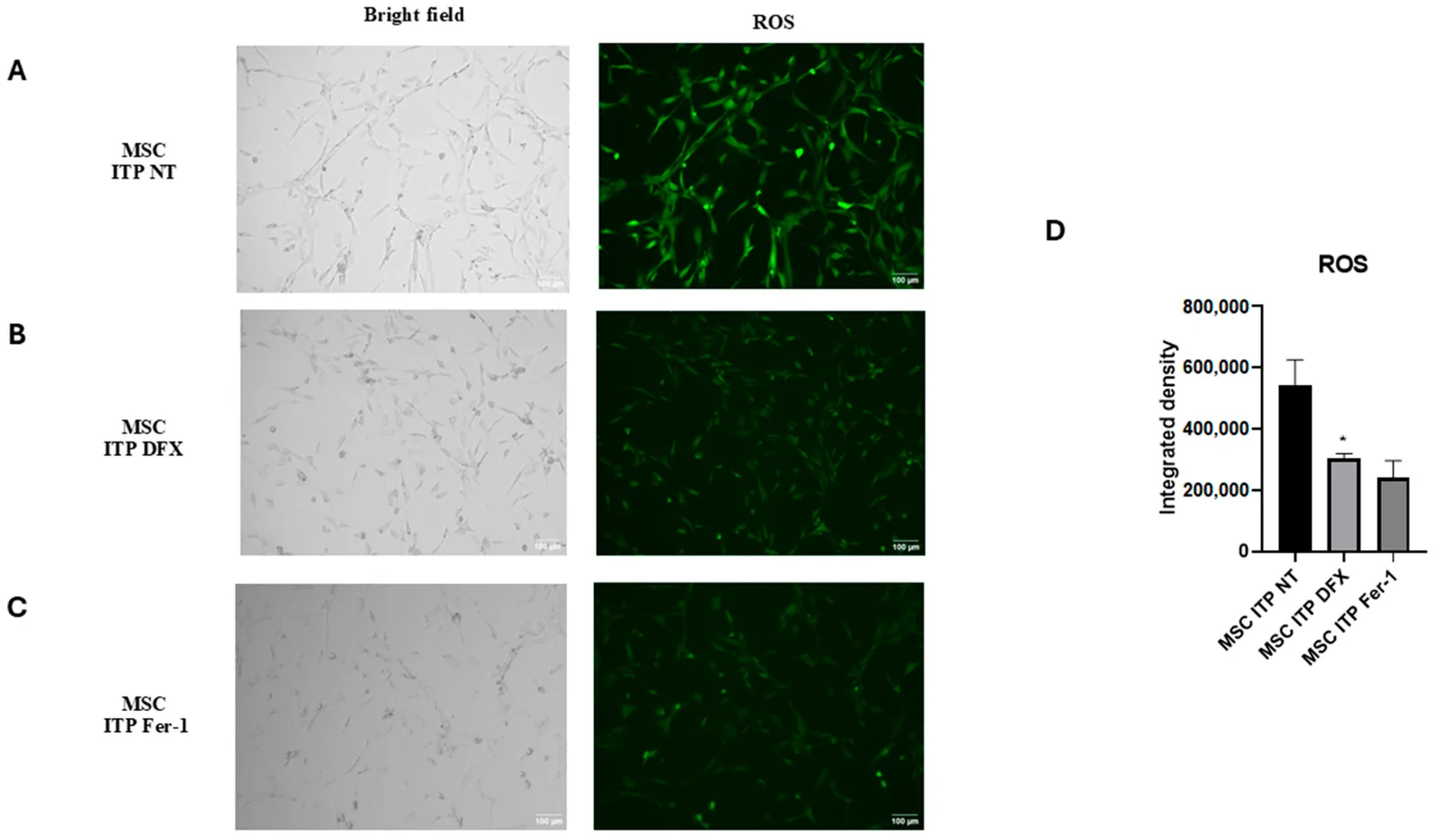
Evaluation of intracellular ROS after Deferasirox and Ferrostatin-1 administration in ITP MSCs. ROS levels in mesenchymal stromal cells (MSCs) isolated from immune thrombocytopenia (ITP) patients before (**A**) and after 24 h exposure to Deferasirox (DFX) [10 µM] (**B**) and Ferrostatin-1 [1 µM] (**C**). The most representative image is displayed. ROS were detected using Thermo Fisher EVOS M5000 Imaging System. ImageJ was used to analyze the images, and integrated density was displayed (**D**). Representative images were acquired using a 10× objective. Scale bar: 100 µm. The graph represents the quantification as mean ± standard deviation (SD) of three independent biological samples per group (N = 3). A *t*-test has been used to evaluate statistical differences. *, *p* = 0.0347 compared to MSC ITP NT.

We evaluated the effects of both DFX and Fer-1 on ROS production in ITP MSCs. DFX [10 μM] and Fer-1 [1 μM] induced a marked reduction in ROS-positive cells compared to untreated cells (Figure 2A–D), suggesting that DFX and Fer-1 may inhibit ferroptosis in ITP, limiting ROS production.

### 2.5. Effect of HD-Dexa on Xc^−^ Antioxidant System

**Figure 3:**
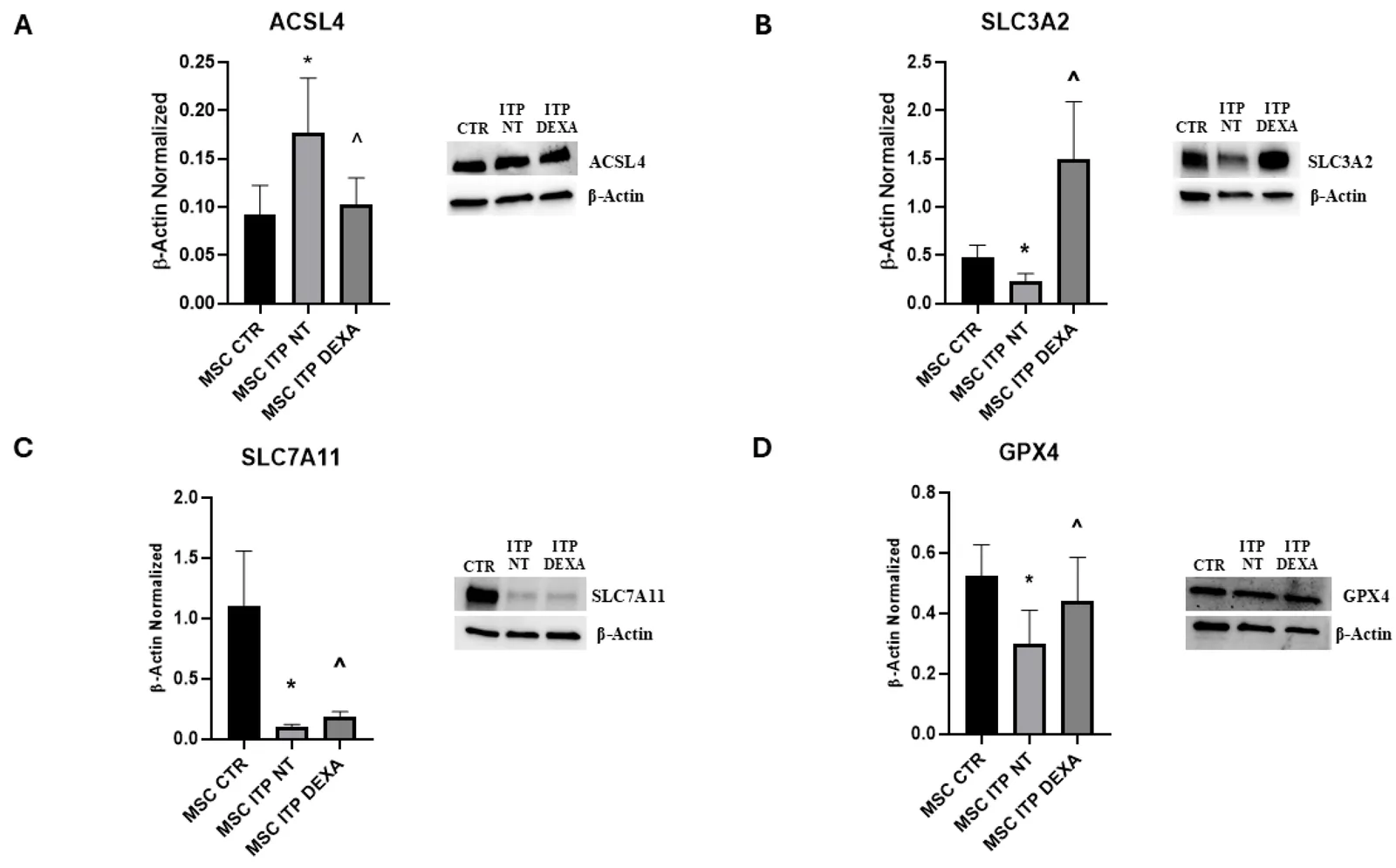
Analysis of ferroptosis-related proteins in CTR and ITP MSCs following High-Dose Dexamethasone treatment. Protein levels of ACSL4 (**A**), SLC3A2 (**B**), SLC7A11 (**C**), and GPX4 (**D**) were assessed by Western blot in mesenchymal stromal cells (MSCs) obtained from control subjects (CTR) and patients with immune thrombocytopenia (ITP). ITP MSCs were analyzed under basal conditions and after 24 h of treatment with 100 nM High-Dose Dexamethasone (HD-Dexa). For each analysis, 15 μg of total protein lysate was loaded. Representative immunoblots are shown. Band intensities were quantified using Image Lab 6.1 software (Bio-Rad, Hercules, CA, USA) and normalized to β-actin as the loading control. Data are reported as mean ± standard deviation (SD) from four independent biological samples per group (N = 4). Statistical significance was evaluated using the *t*-test. * *p* ≤ 0.05 versus CTR MSCs; ^ *p* ≤ 0.05 versus untreated ITP MSCs. Exact *p*-values were as follows: (**A**) ACSL4, ITP NT vs. CTR, *p* = 0.0370; ITP Dexa vs. ITP NT, *p* = 0.0170; (**B**) SLC3A2, ITP NT vs. CTR, *p* = 0.0138; ITP Dexa vs. ITP NT, *p* = 0.0281; (**C**) SLC7A11, ITP NT vs. CTR, *p* = 0.0046; ITP Dexa vs. ITP NT, *p* = 0.0076; (**D**) GPX4, ITP NT vs. CTR, *p* = 0.0246; ITP Dexa vs. ITP NT, *p* = 0.0320.

Performing Western blot of ferroptosis we observed a marked upregulation of ACSL4 protein expression in ITP NT-MSCs compared to CTR-MSCs (Figure 3A), accompanied by a statistically significant downregulation of the cystine transporters SLC3A2 and SLC7A11 (Figure 3B,C), as well as the antioxidant enzyme GPX4 (Figure 3D). These findings suggest that antioxidant defense System Xc^−^ dysfunction may be a cause of ROS accumulation and of ferroptosis induction in ITP-MSCs.

Treatment with HD-Dexa [100 nM] led to a statistically significant reduction in ACSL4 expression and a concomitant increase in SLC3A2, SLC7A11, and GPX4 levels in ITP-MSCs.

### 2.6. Effect of DFX and Fer-1 on Xc^−^ Antioxidant System

**Figure 4:**
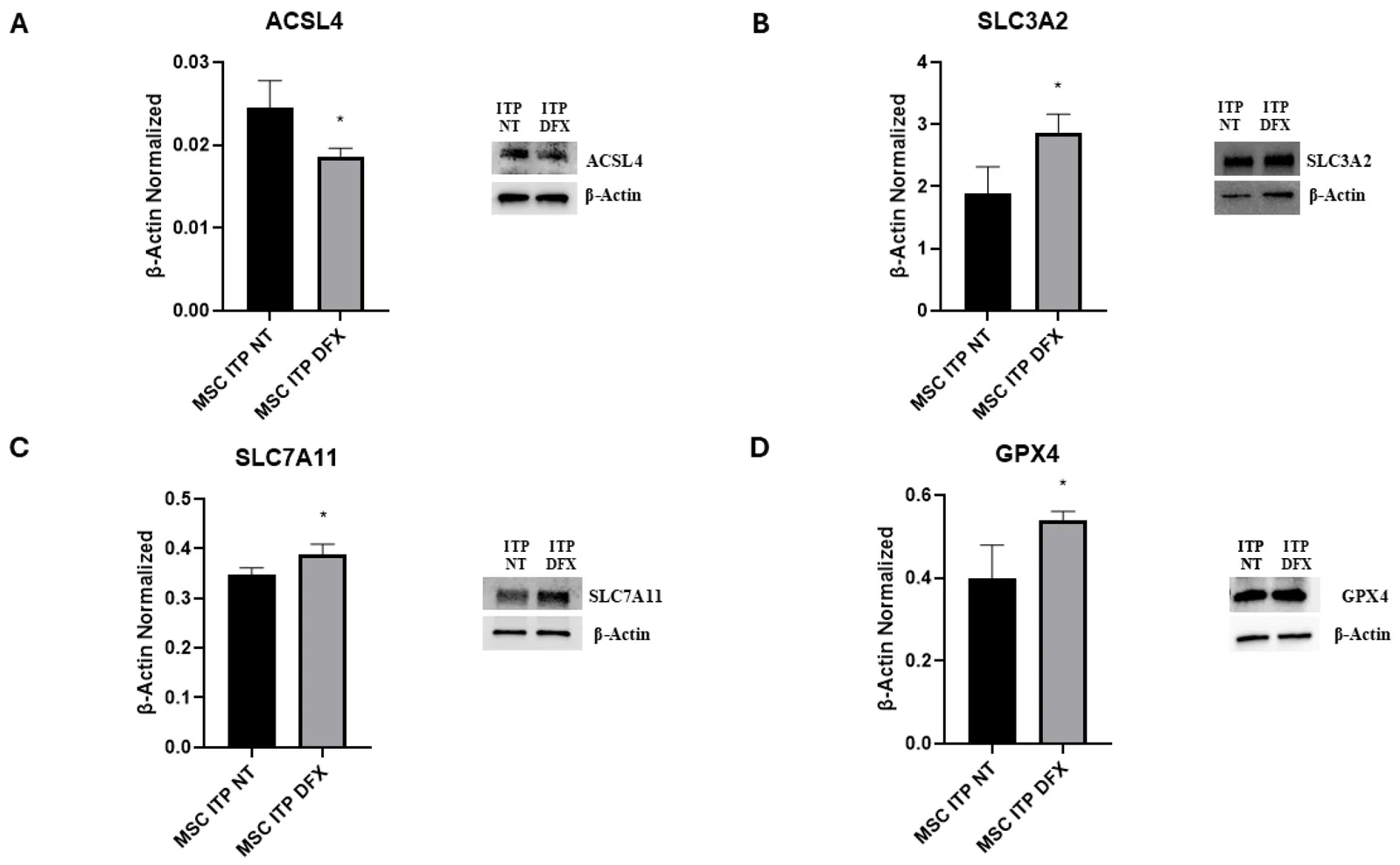
Analysis of ferroptosis-related proteins in ITP MSCs following Deferasirox treatment. Protein levels of ACSL4 (**A**), SLC3A2 (**B**), SLC7A11 (**C**), and GPX4 (**D**) were evaluated by Western blot in mesenchymal stromal cells (MSCs) isolated from patients with immune thrombocytopenia (ITP), before and after 24 h of treatment with 10 µM Deferasirox (DFX). For each analysis, 15 μg of total protein lysate was loaded. Representative immunoblots are shown. Band intensities were quantified using Image Lab 6.1 software (Bio-Rad) and normalized to β-actin as the loading control. Data are presented as mean ± standard deviation (SD) from four independent biological samples (N = 4). Statistical significance was assessed using the *t*-test. * *p* ≤ 0.05 versus untreated ITP MSCs. Exact *p*-values were as follows: (**A**) ACSL4, ITP DFX vs. ITP NT, *p* = 0.0389; (**B**) SLC3A2, ITP DFX vs. ITP NT, *p* = 0.0115; (**C**) SLC7A11, ITP DFX vs. ITP NT, *p* = 0.0201; (**D**) GPX4, ITP DFX vs. ITP NT, *p* = 0.0430.

**Figure 5:**
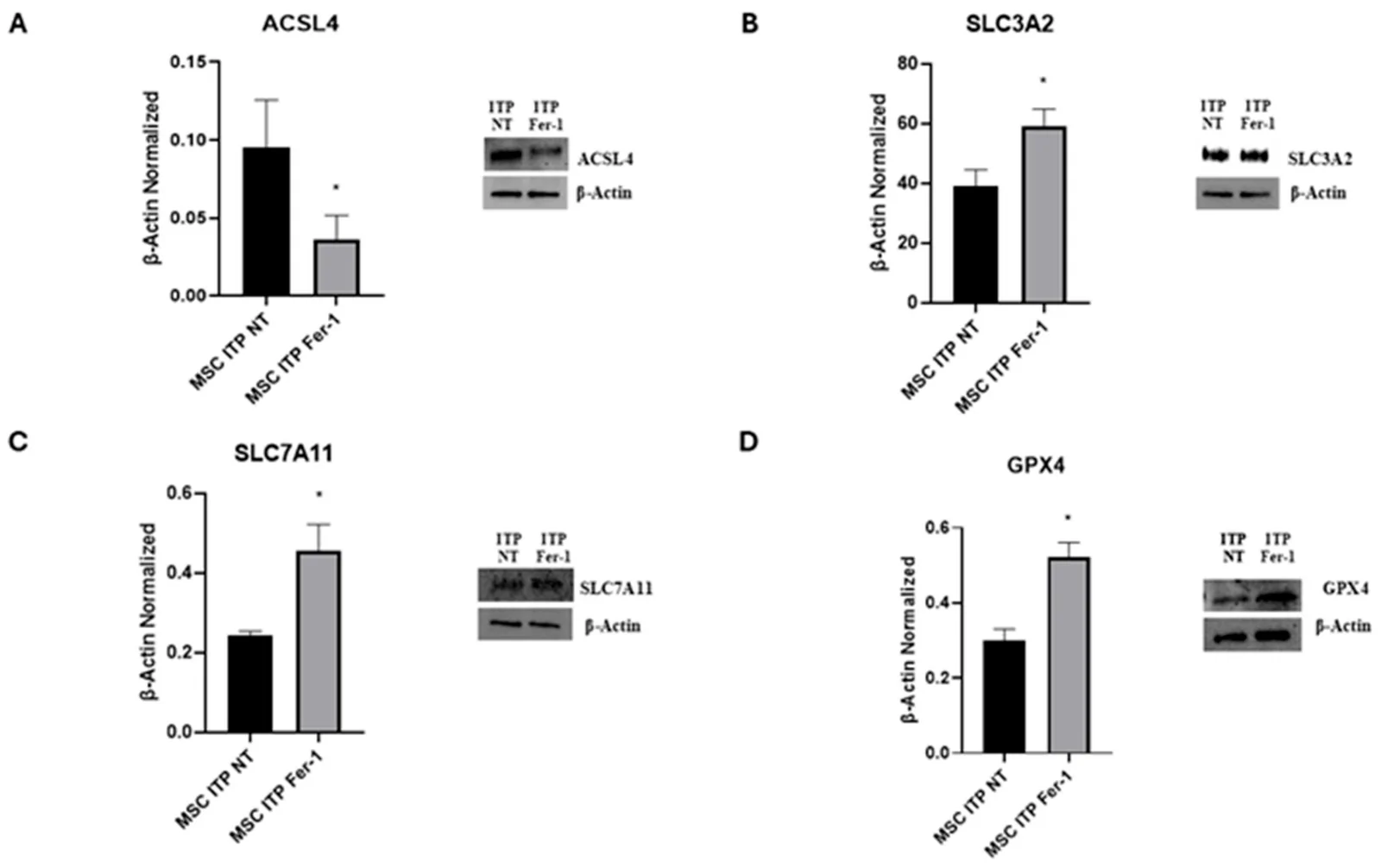
Analysis of ferroptosis-related proteins in ITP MSCs following Ferrostatin-1 treatment. Protein levels of ACSL4 (**A**), SLC3A2 (**B**), SLC7A11 (**C**), and GPX4 (**D**) were evaluated by Western blot in mesenchymal stromal cells (MSCs) isolated from patients with immune thrombocytopenia (ITP), before and after 24 h of treatment with 1 µM Ferrostatin-1 (Fer-1). For each analysis, 15 μg of total protein lysate was loaded. Representative immunoblots are shown. Band intensities were quantified using Image Lab 6.1 software (Bio-Rad) and normalized to β-actin as the loading control. Data are presented as mean ± standard deviation (SD) from four independent biological samples (N = 4). Statistical significance was assessed using the *t*-test. * *p* ≤ 0.05 versus untreated ITP MSCs. Exact *p*-values were as follows: (**A**) ACSL4, ITP Fer-1 vs. ITP NT, *p* = 0.0237; (**B**) SLC3A2, ITP Fer-1 vs. ITP NT, *p* = 0.0154; (**C**) SLC7A11, ITP Fer-1 vs. ITP NT, *p* = 0.0378; (**D**) GPX4, ITP Fer-1 vs. ITP NT, *p* = 0.0061.

We investigated the effect of DFX [10 μM] and Fer-1 [1 μM] on the key components of Xc^−^ antioxidant System. DFX and Fer-1 significantly reduced ACSL4 levels compared to NT ITP-MSC (Figure 4A and Figure 5A). Moreover, both compounds induced a statistically significant increase in SLC3A2 and SLC7A11 transporters (Figure 4B,C and Figure 5B,C), and of GPX4 protein expression levels (Figure 4D and Figure 5D). These results support the protective role of the ferroptosis inhibitors DFX and Fer-1 against ferroptosis.

### 2.7. Effect of HD-Dexa on HMGB-1 Production and Release

**Figure 6:**
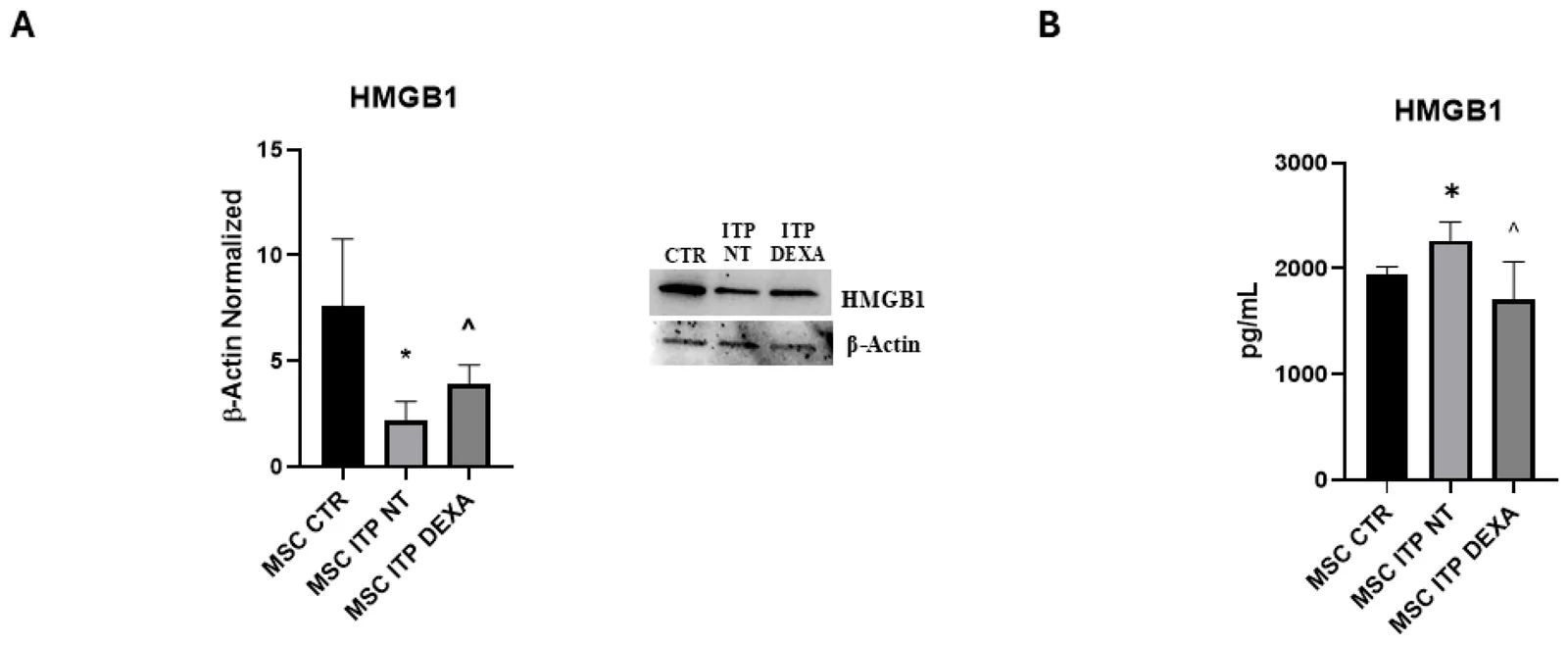
Analysis of intracellular and extracellular HMGB1 levels in CTR and ITP MSCs following High-Dose Dexamethasone treatment. (**A**) Intracellular HMGB1 protein levels were evaluated by Western blot in mesenchymal stromal cells (MSCs) isolated from control subjects (CTR) and patients with immune thrombocytopenia (ITP), under basal conditions and after 24 h of treatment with 100 nM High-Dose Dexamethasone (HD-Dexa). For each analysis, 15 μg of total protein lysate was loaded. Representative immunoblots are shown. Band intensities were quantified using Image Lab 6.1 software (Bio-Rad) and normalized to β-actin as the loading control. Data are presented as mean ± standard deviation (SD) from three independent biological samples per group (N = 3). (**B**) Extracellular HMGB1 levels were measured by enzyme-linked immunosorbent assay (ELISA) in culture supernatants collected from CTR and ITP MSCs before and after 24 h of exposure to 100 nM HD-Dexa. Data are presented as mean ± standard deviation (SD) from four independent biological samples per group (N = 4). Statistical significance was assessed using the *t*-test. * *p* ≤ 0.05 versus CTR MSCs; ^ *p* ≤ 0.05 versus untreated ITP MSCs. Exact *p*-values were as follows: (**A**) ITP NT vs. CTR, *p* = 0.0475; ITP Dexa vs. ITP NT, *p* = 0.0005. (**B**) ITP NT vs. CTR, *p* = 0.0156; ITP Dexa vs. ITP NT, *p* = 0.0183.

We evaluated intracellular HMGB1 protein expression and its extracellular release by Western blot and ELISA, respectively. A marked reduction in HMGB1 expression levels (Figure 6A) and a statistically significant increase in HMGB1 release were observed in ITP NT-MSCs compared to CTR-MSCs (Figure 6B). We demonstrated that HD-Dexa [100 nM] increases HMGB1 protein expression levels compared to ITP NT-MSCs (Figure 6A), and significantly reduces its extracellular release (Figure 6B).

### 2.8. Effect of DFX and Fer-1 on HMGB-1 Production

**Figure 7:**
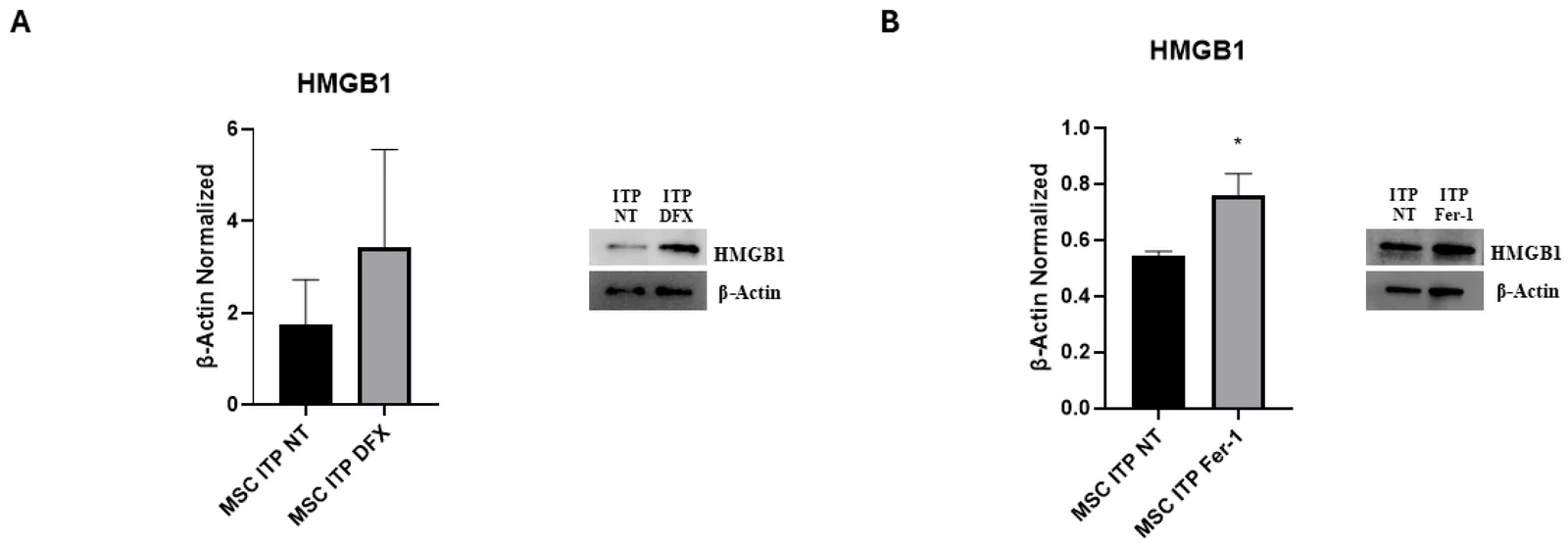
Analysis of intracellular HMGB1 levels in ITP MSCs following Deferasirox or Ferrostatin-1 treatment. HMGB1 protein levels were evaluated by Western blot in mesenchymal stromal cells (MSCs) isolated from patients with immune thrombocytopenia (ITP), before and after 24 h of treatment with 10 µM Deferasirox (DFX) (**A**) or 1 µM Ferrostatin-1 (Fer-1) (**B**). For each analysis, 15 μg of total protein lysate was loaded. Representative immunoblots are shown. Band intensities were quantified using Image Lab 6.1 software (Bio-Rad) and normalized to β-actin as the loading control. Data are presented as mean ± standard deviation (SD) from three independent biological samples (N = 3). Statistical significance was assessed using the *t*-test. * *p* ≤ 0.05 versus untreated ITP MSCs. Exact *p*-values were as follows: (**A**) ITP DFX vs. ITP NT, *p* = 0.1314; (**B**) ITP Fer-1 vs. ITP NT, *p* = 0.0246.

By Western blotting, we observed a trend toward increased intracellular HMGB1 protein expression levels following DFX treatment and a statistically significant increase in its expression after Fer-1 administration (Figure 7A,B). These findings indicate that DFX and Fer-1 modulate intracellular HMGB1 levels in ITP-MSCs.

## 3. Discussion

ITP is the most common pediatric thrombocytopenia caused by autoantibody-mediated platelet destruction [1,2,3,4] and characterized by an increased Th1/Th2 balance, a predominance of pro-inflammatory M1 macrophages and impaired MSCs viability and functions [1,2,4,9,10,11]. MSCs are essential regulators of immune and inflammatory responses, and their dysfunction in ITP represents a critical aspect of disease pathophysiology.

In 2022, our research group demonstrated a dysregulation of iron metabolism in ITP-MSCs, characterized by elevated intracellular iron levels caused by an inflammatory disruption of the hepcidin-FPN1 axis and increased expression of iron importers (TfR1 and DMT1) [2], suggesting a link between iron overload and MSC functional impairment. Here, we investigated whether the previously described iron dysregulation in ITP-MSCs was associated with oxidative stress and molecular alterations involving ferroptosis-related pathways.

Our findings support this hypothesis. ITP-MSCs displayed significantly elevated levels of reactive oxygen species (ROS), indicating marked oxidative stress conditions. We revealed a marked upregulation of ACSL4, crucial for PUFA esterification into membrane phospholipids, and therefore associated with cellular susceptibility to lipid peroxidation [21,23,24]. Conversely, GPX4, SLC7A11 and SLC3A2 were downregulated at the protein level, suggesting an impairment of pathways that are critically involved in protection against ferroptosis [17,18,24,28]. These results collectively indicate that ITP-MSCs exhibit an altered redox state and a molecular profile associated with increased susceptibility to ferroptosis. Although direct assessment of lipid peroxidation and ferroptosis-dependent cell death was not performed, the observed molecular changes are consistent with a ferroptosis-associated phenotype and suggest increased susceptibility to ferroptotic stress in ITP-MSCs.

We also analyzed HMGB1 levels, a DAMP released during ferroptosis as well as other forms of regulated cell death [29,30]. ITP-MSCs showed reduced intracellular HMGB1 protein levels and significantly increased extracellular release, consistent with HMGB1 release from damaged cells. Since extracellular HMGB1 contributes to immune and inflammatory responses, its increased release by ITP-MSCs may further exacerbate the inflammation in ITP. Taken together with the observed changes in ROS and ferroptosis-related proteins, these findings support the presence of a cellular stress phenotype involving ferroptosis-related pathways and suggest an increased susceptibility of ITP-MSCs to ferroptotic stress.

We also explored the effect of HD-Dexa, a corticosteroid frequently used in first-line ITP therapy. Previously, Rossi et al. demonstrated that HD-Dexa, particularly in combination with CB2 stimulation, can restore MSC immunomodulatory function [4]. Our results showed that HD-Dexa modulated oxidative stress and ferroptosis-related molecular alterations in ITP-MSCs. Specifically, HD-Dexa significantly reduced ROS production and ACSL4 expression while increasing SLC3A2, SLC7A11, and GPX4 expression. These molecular changes suggest a restoration of the antioxidant defense system. Moreover, HD-Dexa decreased the extracellular release of HMGB1 and increased its intracellular levels, consistent with reduced cellular stress and DAMP-mediated inflammatory signaling.

Moreover, we investigated the effects of DFX and Fer-1, demonstrating that both compounds modulated oxidative stress and ferroptosis-related molecular alterations. Particularly, DFX and Fer-1 induced a pronounced decrease in ACSL4 protein expression levels and markedly reduced ROS-positive cells. These findings are consistent with modulation of ferroptosis-related pathways in ITP-MSCs.

Their administration also led to an increase in SLC3A2, SLC7A11, GPX4, and intracellular HMGB1 protein expression. Although the changes observed for some individual proteins, such as ACSL4 and SLC7A11, were relatively limited, their biological relevance should be considered within the overall cellular response. These changes occurred together with the modulation of other ferroptosis-related proteins and with a reduction in intracellular ROS following DFX treatment. Thus, even limited variations in individual components may contribute to the overall balance between oxidative stress and antioxidant defense. Altogether, these findings indicate that DFX and Fer-1 modulate multiple molecular pathways associated with ferroptotic stress in ITP-MSCse.

Our data identify increased susceptibility to ferroptotic stress as a novel feature of MSCs from pediatric patients with ITP and show that HD-Dexa, as well as DFX and Fer-1, can modulate oxidative stress and ferroptosis-related molecular alterations. These findings offer new insights into ITP pathophysiology and suggest that targeting ferroptosis-related pathways may represent a potential strategy to limit oxidative damage in ITP-MSCs.

Further in vitro and in vivo studies are needed to validate our findings and better elucidate whether the ferroptosis-associated molecular alterations observed in ITP-MSCs ultimately result in ferroptotic cell death, and to explore the modulation of these pathways as a potential therapeutic strategy.

## 4. Materials and Methods

### 4.1. Source of MSCs

MSCs were isolated from bone marrow samples of 10 children with newly diagnosed ITP and 10 healthy donors (CTR) of the same age (mean age: 7 ± 3 years). Participants were enrolled at the Department of Woman, Child and General and Specialist Surgery of the University of Campania “Luigi Vanvitelli” and at the Department of Haematology of the Bambino Gesù Paediatric Hospital. Healthy donors were individuals who underwent bone marrow collection for haematopoietic stem cell transplantation to siblings with haematological disorders. Bone marrow aspiration was performed in all ITP patients who had previously received intravenous immunoglobulin (IVIG) without achieving a clinical response. None of these patients had received glucocorticoid therapy before bone marrow aspiration and sample collection. The baseline clinical characteristics of the enrolled ITP patients are reported in Supplementary Table S4.

All procedures were conducted in accordance with the Declaration of Helsinki, applicable national regulations, and were approved by the Ethics Committee of the University of Campania “Luigi Vanvitelli” (protocol no. 0029613/i, 21 November 2024). Written informed consent was obtained from all parents or legal guardians and consent from the children prior to participation.

### 4.2. MSCs Isolation, Culture and Characterization

Bone marrow mononuclear cells were separated by density-gradient centrifugation using Ficoll (1.077 g/mL; Lympholyte, Cedarlane Laboratories Ltd., Uden, The Netherlands). The recovered mononuclear cell fraction was resuspended in Alpha Minimum Essential Medium Eagle (α-MEM; Gibco, Thermo Fisher Scientific, Paisley, UK, Cat. Numb.: 12561056) supplemented with 10% human platelet lysate (STEMCELL Technologies Inc., Vancouver, BC, Canada, Cat. Numb.: 06961), 50 U/mL penicillin, and 50 µg/mL streptomycin. Cells were then seeded in coated T25 culture flasks (Corning Costar, Celbio, Milan, Italy). After 72 h, non-adherent cells were discarded and the adherent ones were maintained at 37 °C in a humidified atmosphere containing 5% CO_2_. Culture medium was renewed every 48 h. Upon reaching approximately 80% confluence, adherent cells were detached and subcultured for further expansion up to passage 6 (P6). All MSC samples from both ITP patients and CTR were analyzed at P6 to ensure comparable experimental conditions between groups.

At P6, the MSC immunophenotype was characterized by flow cytometry using FITC- or PE-conjugated monoclonal antibodies directed against HLA-DR, HLA-ABC, CD45, CD34, CD13, CD14, CD31, CD80, and CD90 (BD PharMingen, San Diego, CA, USA), as well as CD73 and CD105 (Serotec, Kidlington, Oxford, UK). Samples were acquired using a FACSCanto flow cytometer, and flow cytometric data were subsequently analyzed using FACSDiva software v9.2, TreeStar Inc., Ashland, OR, USA.

At P6, MSCs obtained from control subjects (CTR-MSCs) and patients with immune thrombocytopenia (ITP-MSCs) were seeded in 24-well plates at a density of 2.5 × 10^4^ cells/well. Cells were exposed for 24 h to high-dose dexamethasone (HD-Dexa; 100 nM), deferasirox (DFX; 10 µM), or ferrostatin-1 (Fer-1; 1 µM). Following treatment, culture supernatants were collected for enzyme-linked immunosorbent assay (ELISA), while cells were harvested and lysed for total protein extraction. For reactive oxygen species (ROS) assessment, P6 MSCs were separately seeded in culture chambers (Falcon™, Corning, NY, USA) and analyzed using a ROS assay (Abcam, Cambridge, UK).

### 4.3. Drugs and Treatments

Dexa (Tocris, Avonmouth, UK, Cat. Numb.: 1126) was dissolved in PBS containing DMSO. Deferasirox (DFX) and Ferrostatin-1 (Fer-1) were purchased from MedChem Express (Cat. Numb.: HI17359 for DFX, HY100579 for FST-1) (distributed by D.B.A., Segrate, Italy) as [10 mM] stock solutions in DMSO. ITP-MSCs were treated for 24 h with Dexa (100 nM), DFX (10 µM), or Fer-1 (1 µM). The final DMSO concentrations in culture were 0.1% for 10 µM DFX and 0.01% for 1 µM Fer-1. Appropriate vehicle controls containing the corresponding DMSO concentration were included for each experimental condition. In the preliminary MTT dose–response experiments, concentration-matched DMSO controls were tested in parallel with DFX, reaching a maximum DMSO concentration of 0.8% at 80 µM DFX. Control cultures (ITP MSCs NT) were maintained in culture medium for the same duration.

### 4.4. MTT Assay

MSCs viability was evaluated after 24 h of DFX and Fer-1 treatments, using the colorimetric 3-(4,5-dimethylthiazol-2-yl)-2,5-diphenyl tetrazolium bromide (MTT) assay (Elabscience, Houston, TX, USA, Cat. Numb.: E-CK-A341). MSCs were seeded at 2500 cells/well in 96-well plates and allowed to adhere for 24 h. The culture medium was then replaced, and cells were treated for an additional 24 h. Three independent biological samples (N = 3) were analyzed, with each experimental condition tested in duplicate wells (N = 2). The analyzed concentrations were DFX [1 µM, 5 µM, 10 µM, 20 µM, 40 µM, 60 µM, 80 µM] and Fer-1 [1 µM, 5 µM, 10 µM, 20 µM, 40 µM, 60 µM, 80 µM].

Mitochondrial dehydrogenases reduced MTT to formazan. After treatment, cells were incubated with the MTT reagent for 4 h under standard culture conditions. The resulting formazan was dissolved in DMSO according to the manufacturer’s instructions, and absorbance was measured at 570 nm. The absorbance values were used to calculate the cell survival rate (%) according to the manufacturer’s instructions.

### 4.5. ROS Assay

Intracellular ROS generation was assessed in viable cells using a commercially available ROS detection assay (Abcam, Cambridge, UK, Cat. Numb.: ab139476), following the manufacturer’s protocol. Total ROS levels were measured using the Oxidative Stress Detection Reagent, a green fluorescent probe with excitation/emission wavelengths of 490/525 nm. Cells were incubated with the detection reagent for 30-60 min, after which the staining solution was removed and the samples were washed. Fluorescence images were acquired using an EVOS M5000 Imaging System (Thermo Fisher Scientific, Waltham, MA, USA). Images were subsequently analyzed using ImageJ software (Fiji, version 1.52; NIH, Bethesda, MD, USA). Background subtraction was performed using a rolling ball radius of 50 pixels, with the “light background” option deselected. The threshold was adjusted using the Li method, taking into account MSC morphology. Individual cell boundaries were manually delineated, and fluorescence was quantified as integrated density (Area × Mean Gray Value). 

### 4.6. Protein Isolation and Western Blot

Total protein lysates were prepared from CTR-MSCs, untreated ITP-MSCs (ITP-MSCs NT), and ITP-MSCs exposed to dexamethasone (ITP-MSCs Dexa), deferasirox (ITP-MSCs DFX), or ferrostatin-1 (ITP-MSCs Fer-1) using RIPA Lysis and Extraction Buffer (Thermo Fisher Scientific, Waltham, MA, USA), following the manufacturer’s protocol. Protein levels of ACSL4, SLC7A11, SLC3A2, GPX4, and HMGB1 were assessed in whole-cell lysates by Western blotting.

Following protein transfer, nitrocellulose membranes were incubated overnight at 4 °C with the following primary antibodies: anti-ACSL4 (rabbit, 1:1000; Elabscience, TX, USA, Cat. Numb.: E-AB-14661; molecular weight [MW]: 79 kDa), anti-SLC7A11 (rabbit, 1:500; Elabscience, Cat. Numb.: E-AB-93104; MW: 55 kDa), anti-SLC3A2 (rabbit, 1:500; Elabscience, Cat. Numb.: E-AB-18594; MW: 68 kDa), anti-GPX4 (rabbit, 1:500; Elabscience, Cat. Numb.: E-AB-67390; MW: 22 kDa), and anti-HMGB1 (rabbit, 1:500; Elabscience, Cat. Numb.: E-AB-15716; MW: 25 kDa). Immunoreactive bands were developed using Clarity Max Western ECL Substrate (Bio-Rad, Hercules, CA, USA) and visualized with a ChemiDoc imaging system (Bio-Rad). β-Actin (mouse monoclonal, 1:500; Santa Cruz Biotechnology, TX, USA, Cat. Numb.: sc-47778; MW: 42 kDa) was used as the loading control.

Images were acquired and stored, and band densitometry was performed using Image Lab 6.1 software (Bio-Rad). Uncropped Western blot images are provided in the Supplementary Material.

### 4.7. ELISA

HMGB1 levels in cell culture supernatants were revealed by an ELISA (Elabscience, TX, USA, Cat. Numb.: E-EL-H1554). Standard solutions, as well as supernatants from ITP-MSCs NT and ITP-MSCs Dexa, were dispensed into wells in duplicate. Following washing, wells were incubated with an enzyme-conjugated polyclonal antibody specific for HMGB1. Subsequent addition of the substrate solution enabled signal development, and absorbance was measured at 450 nm using a DAS Italy microplate reader (DAS Italy, Palombara Sabina, Italy). HMGB1 concentrations (pg/mL) were calculated using the standard curve.

### 4.8. Statistical Analysis

Data distribution was assessed using the Shapiro–Wilk test to evaluate normality. For datasets showing a normal distribution, statistical comparisons were performed using parametric tests: unpaired two-tailed Student’s *t*-test was applied to compare CTR-MSCs and ITP-MSCs NT groups, while paired two-tailed Student’s *t*-test was used to evaluate differences between ITP-MSCs NT and ITP-MSCs Dexa, ITP-MSCs NT and ITP-MSCs DFX, ITP-MSCs NT and ITP-MSCs Fer-1. When data did not follow a normal distribution (SLC7A11 expression in CTR samples), non-parametric analysis was conducted using the Mann–Whitney U test for unpaired comparisons between CTR-MSCs and ITP-MSCs NT. All analyses were performed using GraphPad Prism version 8.4.2. Results were considered statistically significant at *p* < 0.05. N denotes the number of independent biological replicates, each corresponding to an independent primary MSC culture obtained from a different donor. The specific number of biological replicates used for each experiment (N = 3 or N = 4) is reported in the corresponding figure legends.

## Data Availability

The raw data supporting the conclusions of this article will be made available by the authors on request.

## Supplementary Materials

The following supporting information can be downloaded at: https://www.mdpi.com/article/10.3390/ijms27198917/s1. Reference [31] has been cited in the Supplementary Materials.

## Abbreviations

ITP	Immune Thrombocytopenia
MSCs	Mesenchymal stromal cells
Fer-1	Ferrostatin-1
DFX	Deferasirox
HD-Dexa	High-dose dexamethasone
Th	T helper
FPN1	Ferroportin
TfR1	Transferrin receptor 1
DMT1	Divalent metal transporter 1
ROS	Reactive oxygen species
PUFAs	Polyunsaturated fatty acids
ACSL4	Acyl-CoA synthetase long-chain family member 4
GSH	Glutathione
GPX4	Glutathione peroxidases 4
DAMPs	Damage-associated molecular patterns
HMGB1	High-mobility group box 1
CB2	Cannabinoid receptor 2
CTR-MSCs	MSCs derived from healthy donors
MTT	3-(4,5-dimethylthiazol-2-yl)-2,5-diphenyl tetrazolium bromide
