# Supplementary material for: Ferroptosis Susceptibility in Pediatric Immune Thrombocytopenia: Alteration in Mesenchymal Stromal Cells
Source: Int J Mol Sci. 2026 Oct 8;27(19):8917. doi: 10.3390/ijms27198917 (PMC13665037; doi:10.3390/ijms27198917)
Supplement: Supplementary file 1 [file ijms-27-08917-s001.zip › Supplementary Table S1.pdf]

	Positivity (%)	
	**CTR-MSCs**	**ITP-MSCs**
**CD105**	100	100
**CD73**	100	100
**CD90**	100	100
**HLA-ABC**	97,10	98,90
**CD45**	0,06	0,13
**CD14**	0,19	0,31
**CD34**	0,46	0,50
**CD13**	0,16	0,20
**CD31**	0,04	0,11
**CD80**	0,47	0,29
**HLA-DR**	0,11	0,24

Supplementary Table S1. Immunophenotypic characterization of mesenchymal stromal cells (MSCs) derived from healthy donors (CTR-MSCs) and patients with immune thrombocytopenia (ITP-MSCs). The table reports the surface antigens detected by flow cytometric analysis using a FACSCanto cytometer (BD Biosciences, Milan, Italy), along with the corresponding percentage of positive cells.
