# Supplementary material for: Ferroptosis Susceptibility in Pediatric Immune Thrombocytopenia: Alteration in Mesenchymal Stromal Cells
Source: Int J Mol Sci. 2026 Oct 8;27(19):8917. doi: 10.3390/ijms27198917 (PMC13665037; doi:10.3390/ijms27198917)
Supplement: Supplementary file 1 [file ijms-27-08917-s001.zip › Supplementary Table S2.pdf]

DFX - MTT CELL SURVIVAL RATE (%)	
**ITP-MSC NT**	100±3.7
**ITP-MSC DFX [1 µM]**	99.7±1.1
**ITP-MSC DFX [5 µM]**	99.3±1.8
**ITP-MSC DFX [10 µM]**	92.4±4.0*
**ITP-MSC DFX [20 µM]**	49.0±1.6*
**ITP-MSC DFX [40 µM]**	8.3±1.9*
**ITP-MSC DFX [60 µM]**	3.0±1.3*
**ITP-MSC DFX [80 µM]**	2.3±0.2*

Supplementary Table S2. Effects of Deferasirox (DFX) on survival of ITP-MSC and evaluation of its cytotoxicity. Cell survival rate in Immune Thrombocytopenia (ITP) patients' mesenchymal stromal cells (MSCs) after 24 h of treatment with Deferasirox (DFX) at different concentrations (1 µM, 5 µM, 10 µM, 20 µM, 40 µM, 60 µM, and 80 µM) were evaluated by performing an MTT assay. The results are presented as the mean percentage ± standard deviation percentage (SD). Student's t-test was used for statistical analysis. * $p \leq 0.05$ compared to non-treated (NT) MSCs. Exact p-values for significant comparisons were as follows: DFX 10 µM vs. NT, p = 0,0016; DFX 20 µM vs. NT, p = 0,0010; DFX 40 µM vs. NT, p = 0,0001; DFX 60 µM vs. NT, p = 0,0006; DFX 80 µM vs. NT, p = 0,0004.
