# Supplementary material for: Ferroptosis Susceptibility in Pediatric Immune Thrombocytopenia: Alteration in Mesenchymal Stromal Cells
Source: Int J Mol Sci. 2026 Oct 8;27(19):8917. doi: 10.3390/ijms27198917 (PMC13665037; doi:10.3390/ijms27198917)
Supplement: Supplementary file 1 [file ijms-27-08917-s001.zip › Supplementary Table S3.pdf]

Fer-1 - MTT CELL SURVIVAL RATE (%)	
**ITP-MSC NT**	100±6.8
**ITP-MSC Fer-1 [1 µM]**	99.3±3.1
**ITP-MSC Fer-1 [5 µM]**	91.1±10.3
**ITP-MSC Fer-1 [10 µM]**	77.5±9.3
**ITP-MSC Fer-1 [20 µM]**	47.5±8.1*
**ITP-MSC Fer-1 [40 µM]**	12.5±1.6*
**ITP-MSC Fer-1 [60 µM]**	3.8±0.4*
**ITP-MSC Fer-1 [80 µM]**	0.3±0.9*

Supplementary Table S3. Effects of Ferrostatin-1 (Fer-1) on survival of ITP-MSC and evaluation of its cytotoxicity. Cell survival rate in Immune Thrombocytopenia (ITP) patients' mesenchymal stromal cells (MSCs) after 24 h of treatment with Ferrostatin-1 (Fer-1) at different concentrations (1 µM, 5 µM, 10 µM, 20 µM, 40 µM, 60 µM, and 80 µM) were evaluated by performing an MTT assay. The results are presented as the mean percentage ± standard deviation percentage (SD). Student's t-test was used for statistical analysis. * $p \leq 0.05$ compared to non-treated (NT) MSCs. Exact p-values were as follows: Fer-1 20 µM vs. NT, p = 0,0200; 40 µM vs. NT, p = 0,0013; 60 µM vs. NT, p = 0,0021; 80 µM vs. NT, p = 0,0006.
