# Supplementary material for: Ferroptosis Susceptibility in Pediatric Immune Thrombocytopenia: Alteration in Mesenchymal Stromal Cells
Source: Int J Mol Sci. 2026 Oct 8;27(19):8917. doi: 10.3390/ijms27198917 (PMC13665037; doi:10.3390/ijms27198917)
Supplement: Supplementary file 1 [file ijms-27-08917-s001.zip › Supplementary Table S4.pdf]

ITP Patients	
**Median age, years (mean ± SD, years)**	7 ± 3
**Sex (Female/Male)**	4/6
**Platelet count (range, × 10^9/L)**	10-35
**Disease Duration (range, days)**	20-90
**Infection (Yes/No)**	No
**Vaccination (Yes/No)**	No
**Ferritin (mean ± SD, ng/mL)**	51.5 ± 16.8
**Transferrin saturation (mean ± SD, %)**	29.1 ± 7.0
**CRP (mean ± SD, mg/dL)**	0.03 ± 0.01
**Glucocorticoids (Yes/No)**	No
**Without Fatigue (n/10)**	10/10
**Without family history of autoimmune disease (n/10)**	10/10
**Bleeding grade (Buchanan & Adix)***	≥ 3

Supplementary Table S4. Clinical characteristics of ITP patients. This table shows the clinical characteristics of 10 enrolled ITP patients in the study. *Bleeding severity was classified according to the Buchanan & Adix score, as recommended by the AIEOP Consensus [31].
